# Lactation-Related MicroRNA Expression Profiles of Porcine Breast Milk Exosomes

**DOI:** 10.1371/journal.pone.0043691

**Published:** 2012-08-24

**Authors:** Yiren Gu, Mingzhou Li, Tao Wang, Yan Liang, Zhijun Zhong, Xiaoyan Wang, Qi Zhou, Lei Chen, Qiulei Lang, Zhiping He, Xiaohui Chen, Jianjun Gong, Xiaolian Gao, Xuewei Li, Xuebin Lv

**Affiliations:** 1 Sichuan Animal Science Academy, Chengdu, Sichuan, China; 2 Institute of Animal Genetics & Breeding, College of Animal Science & Technology, Sichuan Agricultural University, Ya’an, Sichuan, China; 3 Peking-Tsinghua Center for Life Sciences, Biodynamic Optical Imaging Center & College of Life Sciences, Peking University, Beijing, China; 4 Department of Nursing, Ya’an Vocational College, Ya’an, Sichuan, China; 5 Chongqing Academy of Animal Science, Chongqing, China; 6 LC-Bio, Hangzhou, Zhejiang, China; 7 Department of Biology & Biochemistry, University of Houston, Houston, Texas, United States of America; UMDNJ-New Jersey Medical School, United States of America

## Abstract

Breast milk is the primary source of nutrition for newborns, and is rich in immunological components. MicroRNAs (miRNAs) are present in various body fluids and are selectively packaged inside the exosomes, a type of membrane vesicles, secreted by most cell types. These exosomal miRNAs could be actively delivered into recipient cells, and could regulate target gene expression and recipient cell function. Here, we analyzed the lactation-related miRNA expression profiles in porcine milk exosomes across the entire lactation period (newborn to 28 days after birth) by a deep sequencing. We found that immune-related miRNAs are present and enriched in breast milk exosomes (*p*<10^−16^, *χ* ^2^ test) and are generally resistant to relatively harsh conditions. Notably, these exosomal miRNAs are present in higher numbers in the colostrums than in mature milk. It was higher in the serum of colostrum-only fed piglets compared with the mature milk-only fed piglets. These immune-related miRNA-loaded exosomes in breast milk may be transferred into the infant body via the digestive tract. These observations are a prelude to in-depth investigations of the essential roles of breast milk in the development of the infant’s immune system.

## Introduction

Breast milk is the milk produced by the mammary glands of a female mammal for the infant offspring, and contains a balance of nutrients that closely matches infant requirements for brain development, growth, and a healthy immune system, which provides a distinct advantage over formula [1], [2]. Compared with mature milk, the initial milk (usually 0 to 3 days after birth) is often referred to as colostrum, and is higher in immunological agents and other compounds that act against viruses, bacteria, and parasites [3]. This helps to protect the newborn until its own immune system can function properly [4].

MicroRNAs (miRNAs), an abundant class of evolutionarily conserved small non-coding RNAs of ∼22 nucleotides (nt) that are derived from 70 nt long stem-loop precursors (pre-miRNAs), are post-transcriptional regulators that bind to complementary sequences on target mRNAs, usually resulting in translational repression in mammals [5], [6]. Emerging evidence suggests that miRNAs have important roles in regulating the development of immune cells and in modulating innate and adaptive immune responses [7]. Extracellular miRNAs in various body fluids (such as amniotic fluid, breast milk, blood, bronchial lavage, malignant ascites fluid, tears, saliva, and urine) have recently been shown to be associated with various pathological conditions [8]. These circulating miRNAs are mainly delivered by exosomes, which are membranous vesicles (30–100 nm in diameter) of endocytic origin that are released by a variety of cell types into the extracellular space [9]. Exosomes appear to play a significant role in cellular communication in the immune system and elsewhere by transferring miRNAs [10], [11], mRNAs [12], and proteins [13] to neighboring cells. Exosomes are present in human breast milk and are packaged with abundant immune-related proteins (such as MHC class II, CD86, and the tetraspanin proteins, CD63 and CD81) [13], as well as miRNAs, have the potential to influence the immune system of the infant [10], [11].

Here, we present the miRNA expression profiles in porcine milk exosomes across six lactigenous stages (0, 3, 7, 14, 21, and 28 days after birth, hereafter referred to as 0d, 3d, 7d, 14d, 21d, and 28d, respectively). We particularly emphasize the differences between the colostrum (initial milk, usually 0 to 3 days after birth) and mature milk (later milk, 7 to 28 days after birth). We found that the colostrum contains more abundant immunomodulatory features (such as exosomal immune-related miRNAs) compared with mature milk, and may have significant effects on the development of the immune system in infants. This study also highlights the use of pigs as a model organism for human breast-feeding medicine and immune diseases research.

## Results

### miRNAs-loaded Exosomes are Present in Breast Milk

**Figure 1:**
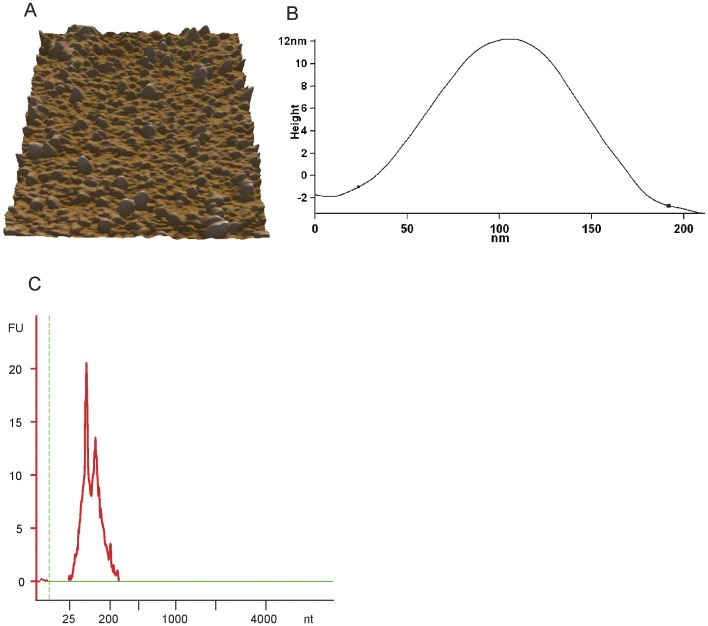
Evidence of miRNA-loaded exosomes in porcine breast milk. (A) 3D AFM image of isolated milk exosomes. (B) The line profile of AFM image for a milk exosome. X- and Y-axes are the width and height of a porcine milk exosome, respectively. (C) RNA from porcine breast milk exosomes was detected using Agilent Bioanalyzer 2100.

Exosomes were isolated from porcine breast milk by ultracentrifugation and were investigated using atomic force microscope (AFM) at the nanometer-scale. Porcine breast milk contains a substantial amount of exosome-like vesicles (Fig. 1A) which had an approximate width of 160 nm and a height of 12 nm (Fig. 1B), and exhibited the similar flattened donut-like structures with the exosomes in human saliva and breast milk [11], [14]. We also found that the milk exosomes contain a considerable number of small RNAs that were below 300 nt in length, but few or no 18S and 28S ribosomal RNA (Fig. 1C), which confirmed the enrichment of miRNAs in exosomes.

### Identification of the Exosomal miRNAs in Breast Milk

Each small RNA library generated ∼22.49 million (M) 36-nt reads, resulting in more than 179.90 M raw reads for eight libraries. After remove the low quality reads [15], [16], the remain reads were considered as “mappable reads” for each library, corresponding to ∼0.12 M kinds of reads. The higher counts/kinds ratio (22.83) for the mappable reads ensured a higher number of replicates for a bona fide miRNA-derived molecule and reduced the possibility of sequencing errors. Of these mappable reads in the eight libraries, the overwhelming majority are 21∼24 nt in length (91.97±3.18%). More than half of the reads are 22 nt in length (67.93±4.81%), followed by 23 nt (15.92±2.27%), 24 nt (4.71±1.65%), and 21 nt (3.42±1.77%), which are typical sizes of Dicer-processed products [5], [6] (Fig. S1), confirming the presence of miRNAs in milk exosomes (Fig. 1C).

**Figure 2:**
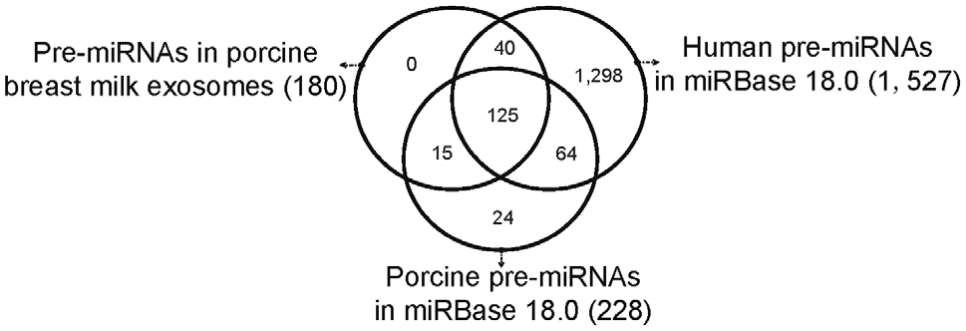
Distribution of shared pre-miRNAs among miRBase (Version 18.0) entries for human (1,527) and pig (228), as well as 180 pre-miRNAs identified in porcine breast milk exosomes. Out of 180 pre-miRNAs in porcine breast milk exosomes, 140 are known porcine pre-miRNAs and 40 are novel porcine pre-miRNAs, which encoding 237 mature miRNAs, corresponding to 234 unique miRNAs.

Analysis of the mapped pre-miRNAs indicates that 180 pre-miRNAs were detected in the eight exosomal small RNA libraries (Fig. 2), of which 140 (77.78%) are known porcine pre-miRNAs and 40 are novel porcine pre-miRNAs. These 40 novel porcine pre-miRNAs are not mapped to known porcine pre-miRNAs, but are homologous to human pre-miRNAs and could be mapped to the pig genome. Notably, out of all 228 known porcine pre-miRNAs deposited in miRBase 18.0, 39 pre-miRNAs are pig-specific and not homologous to human pre-miRNAs. Nonetheless, the 180 pre-miRNAs present in pig breast milk exosomes are all conserved between pig and human, which implies a similar miRNA-regulated physiological and pathological mechanism in breast milk between mammals [17]. Predictably, there are distinct pre-miRNAs and genomic loci that express identical mature sequences, which resulted in these 180 pre-miRNAs encoding 237 mature miRNAs, corresponding to 234 unique miRNAs (Fig. 2).

### Lactation-related miRNA Expression Profiles

**Figure 3:**
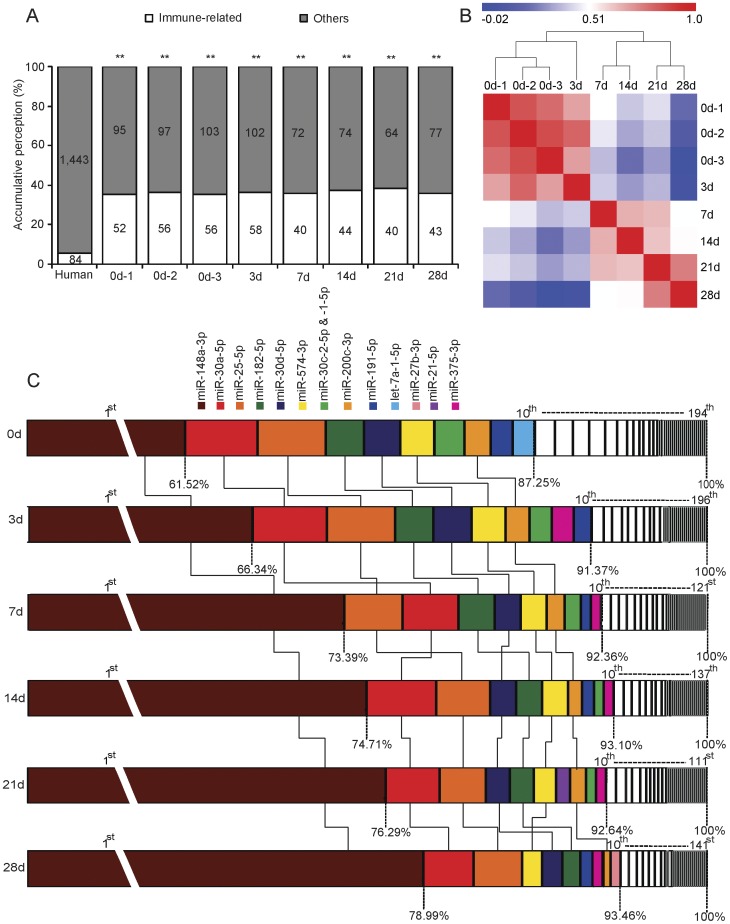
Lactation-related miRNA expression profiles. (A) Distribution of lactation-related pre-miRNAs. Out of 1,527 pre-miRNAs deposited in miRBase 18.0, 84 (5.50%) pre-miRNAs have been designated as immunopathology-related pre-miRNAs, based on annotation in the Pathway Central database (SABiosciences, MD, USA). These immune-related pre-miRNAs are enriched in each milk exosomal miRNA library. *χ* ^2^ test (**p<10^−16^): Numbers of immunopathology-related miRNAs and others detected in milk exosomes compared with the total entries in miRBase 18.0. The three biological replicates at 0 days are denoted by 0d-1, -2 and -3, respectively. (B) Hierarchical clustering and heat map matrix of pairwise Spearman correlations of the counts of 234 unique miRNAs between eight exosomal miRNA libraries. (C) Top 10 unique miRNAs with the highest expression levels in milk exosomal miRNA libraries. Plot of the unique miRNAs versus their % in total counts of all unique miRNAs for each library. The dashed vertical lines represent the accumulative % of the top 10 unique miRNAs in total counts of all unique miRNAs. Seven miRNAs that are present in the top 10 miRNAs in all six libraries are connected by lines.

Similar to our previous human breast milk report [10], out of 84 immune-related pre-miRNAs that are most relevant to the function of lymphocytes and other immune cells based on the annotation of the Pathway Central database (SABiosciences, MD, USA), 58 (69.05%) are present and enriched in each milk exosomal miRNA library (*p*<10^−16^, *χ* ^2^ test) (Fig. 3A). As shown in Figure 3B, the miRNA expression profiles show an obvious lactation-specific pattern. Two major branches are defined: one representing 0 and 3 days, and one representing 7, 14, 21, and 28 days. This different clustering pattern may correspond to the intrinsically different biochemical and physiological components of porcine colostrum (0 to 3 days after birth) and mature milk (7 to 28 days after birth) [3]. Moreover, the three biological milk replicates at 0 days were highly correlated with each other (average *r* = 0.833), which suggested experimental reliability and further highlighted the low variation in exosomal miRNA profiles in breast milk across different individuals.

In the breast milk exosomal miRNA transcriptome, we observed that the majority of abundant miRNAs are from few miRNAs. As shown in Figure 3C, the top 10 unique miRNAs with the highest expression level account for more than 87.25%, by total counts, of all the 234 unique miRNAs. The unified set of top 10 unique miRNAs over six lactigenous stages corresponds to 13 kinds of unique miRNAs. Among these 13 types of miRNA, seven miRNAs (miR-148a-3p, miR-182-5p, miR-200c-3p, miR-25-3p, miR-30a-5p, miR-30d-5p, and miR-574-3p) are present in the top 10 miRNAs in all six libraries, which suggests essential roles in various immune and pathological conditions.

miR-25-3p, an important member of the miR-106b∼25 cluster, resides in an intronic region of the well-characterized oncogene, *MCM7* (minichromosome maintenance protein 7). miR-25-3p targets the potent mediator of inflammation, *KLF4* (Krüppel-like factor 4) [18] and has essential roles in the development of the immune system. miR-30a-5p and -30d-5p, two members of the miR-30-family, are not only involved in mitochondrial fission by suppressing the expression of *p53* (tumor protein 53) and its downstream target *DRP1* (dynamin-related protein 1), but also promote cellular invasion and immunosuppression by directly targeting *GALNT7* (GalNAc transferase 7), resulting in increased synthesis of the immunosuppressive cytokine *IL-10* (interleukin-10) [19]. miR-182-5p is induced by *IL-2* (interleukin-2) and promotes T cell-mediated immune responses though post-transcriptionally inhibited *FOXO1* (forkhead box protein O1), a suppressor of proliferation expressed in resting helper T lymphocytes [20]. miR-200c-3p targets *ZEB1* (zinc finger E-box-binding homeobox 1) [21], which has been shown to regulate T-cell differentiation, to repress *IL-2* production, and to regulate the expression of *CD4* (cluster of differentiation 4) [22]. miR-574-3p is a potential biomarker for detecting and differentiating the major subtypes of diffuse large B-cell lymphomas [23].

In particular, and similar to a previous report of human [10] and bovine breast milks [24], miR-148a-3p, as a biomarker of primary effusion lymphoma, takes the top ranking across six lactigenous stages (∼0.15 M) by counts, accounting for ∼71.87% (by counts) of the all unique miRNAs. miR-148a-3p directly targets the cancer-related *TGIF2* (TGFB-induced factor homeobox 2) [25] and drug-metabolizing-related *PXR* (pregnane×receptor) genes [26]. Although the underlying regulatory mechanism of the most predominant miRNA (miR-148a-3p) packaged into milk exosomes is still unclear, it is intriguing to suggest that miR-148a-3p is a potential biomarker for the quality control of mammalian milk [10], [24].

In addition, other six miRNAs (let-7a-1-5p, miR-30c-2-5p and -1-5p, miR-191-5p, miR-375-3p, miR-21-5p, and miR-27b-3p) in the unified set of top 10 most highly expressed unique miRNAs over six lactigenous stages are related to various immune and pathological responses. let-7a-1-5p modulates inflammation-associated cytokine *IL-6* (interleukin-6)-induced STAT3 (signal transducers and activators of transcription 3) signaling [27]. miR-30c-2-5p and -1-5p are also members of the miR-30-family and are involved in oncogenesis [20] and immunosuppression [19]. miR-191-5p, a well-characterized oncogenesis-related miRNA, is a biomarker of colorectal cancer [28], primary effusion lymphoma [29], and hepatocellular carcinoma [30]. miR-375-3p, as a key regulator of epithelial properties necessary for securing epithelium–immune system crosstalk, is induced by *IL-13* (interleukin 13) and regulates the expression of *TSLP* (thymic stromal lymphopoietin), an epithelium-derived cytokine [31]. miR-21-5p negatively regulates an innate immune receptor, *TLR4* (toll-like receptor 4) by targeting the proinflammatory tumor suppressor, *PDCD4* (programmed cell death protein 4) [32], as well as directly modulating a key cytokine in immune regulation, *IL-12* (interleukin 12). miR-27b-3p destabilizes the mRNA abundance of lipopolysaccharide-mediated *PPARγ* (peroxisome proliferator-activated receptor *γ*), which is often associated with chronic inflammatory diseases provoked by insufficient resolution of an immune response [33].

These immune-related miRNAs, expressed at high levels throughout the entire porcine lactation period, highlight the important roles of exosomal miRNAs as immune-regulatory agents in milk.

### Expression of Immune-related miRNAs in Exosomes between the Colostrum and Mature Milk

**Figure 4:**
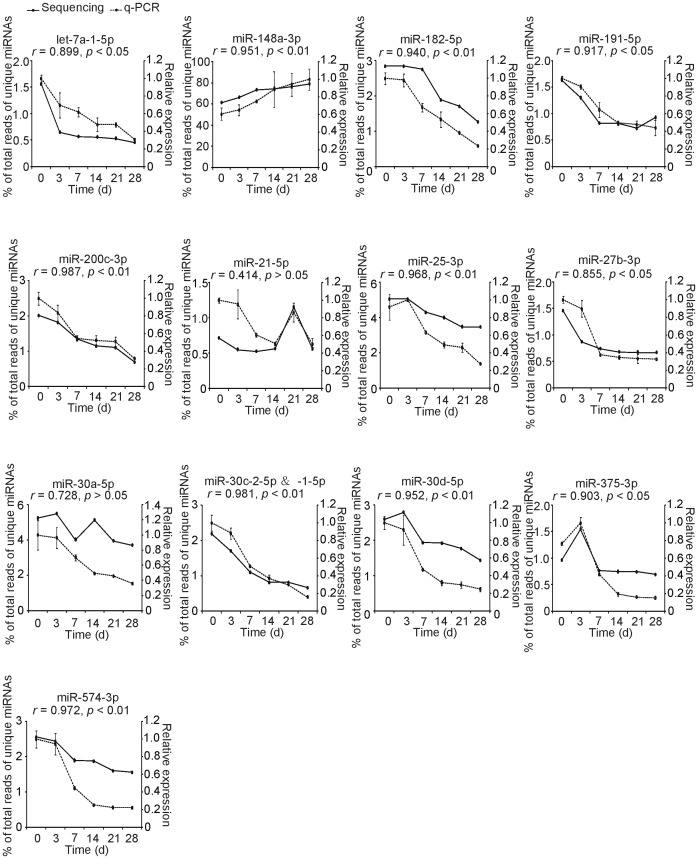
Lactation-related expression patterns of 13 abundant immune-related miRNAs. The data are normally distributed (Kolmogorov-Smirnov test, *p*>0.05). Pearson correlation was used to determine the relation of miRNAs expression changes between the q-PCR and the deep sequencing approaches. Values are means±SD.

We measured the expression patterns of these 13 unique immune-related miRNAs in milk exosomes over six lactigenous stages, using a sensitive quantitative PCR (q-PCR) approach. As shown in Figure 4, apart from miR-148a-3p, almost all the other 12 unique miRNAs exhibited higher abundances in the early lactation periods (0 and 3 days) than in the later lactation periods (7, 14, 21 and 28 days). These 12 immune-related miRNAs have been shown to functionally target specific transcripts encoding cytokines, other immunological regulatory proteins, and immune response signaling pathway components [7]. These 13 unique immune-related miRNAs represent the majority of abundant miRNAs (Fig. 3C) and may characterize the predominant immune-regulatory agents in the breast milk exosomal miRNA transcriptome. This result, derived from nucleic acid changes (i.e. miRNAs), corresponds with the well-established results at the protein level [3]. The early milk (particularly colostrum) is rich in immunoglobulins (particularly IgA) and immunomodulatory cytokines (especially IL-1, IL-6 and TNF-*α*) but lower in fat and caseins. The later mature milk, a rich source of nourishment for infant growth, mainly contains lactose and casein, but with a substantial reduction in immunological compositions.

Interestingly, the expression level of the most abundant miRNA, miR-148a-3p, increased during the lactation periods, which contrasted with the other immune-related miRNAs (Fig. 4). miR-148a-3p is not only correlated with immunopathology, but also directly targets and suppresses the expression of a well-characterized gene, *DNMT3B* (DNA methyltransferase 3b), which encodes a *de novo* methyltransferase that sets up DNA methylation patterns early in development [34]. Although the detailed relationship between the inverse lactation-related expression pattern of miR-148a-3p and other immune-related genes is unclear, it is tempting to speculate that the enrichment of miR-148a-3a in milk exosomes leads to a downregulation of *DNMT3B* and a consequent inactivation of *de novo* methylation in cells of the infant digestive tract.

Furthermore, we also observed a good correlation between the deep sequencing and the q-PCR expression data for these 13 immune-related miRNAs over six lactation stages (Pearson, *r* = 0.882±0.157), which indicated that our deep sequencing results reliably reveal the characteristics of the milk exosomal miRNA transcriptome.

### Resistance to Degradation and Stability of Milk-derived miRNAs

We next sought to survey the stability of miRNAs in breast milk across various harsh conditions, which is a vital prerequisite for our hypothesis that these circulating miRNAs are transferable genetic material from the mother’s milk to the infant via the digestive tract.

Similar to previous studies on blood [35] and human breast milk [10], [11], the exogenous synthetic worm-specific miRNAs were sharply degraded; however, the endogenous milk-derived miRNAs exhibited resistance to degradation when the milk was subjected to prolonged incubation at different temperatures, low pH, multiple freeze-thaw cycles or even treatment with high concentrations of exogenous RNase (Fig. S2). These results suggest that milk-derived miRNAs exist in a remarkable stable form that is protected from endogenous RNase activity across various harsh conditions. The remarkable stability of the endogenous milk-derived miRNAs implies the potential mechanism that the breast milk allows dietary intake of miRNAs by infants.

### Expression of Immune-related miRNAs between Colostrum-only and Mature Milk-only Fed Piglets

It is established that miRNAs are selectively packaged into exosomes and actively delivered into recipient cells where the exogenous miRNAs can regulate target gene expression and recipient cell function [36]. Based on the above results that these immune-related miRNAs are present in higher numbers in the colostrum compared with the mature milk (Fig. 4) and are resistant to general harsh conditions (Fig. S2), we sought to investigate the effects of the dietary intake of milk-derived miRNAs by infants.

**Figure 5:**
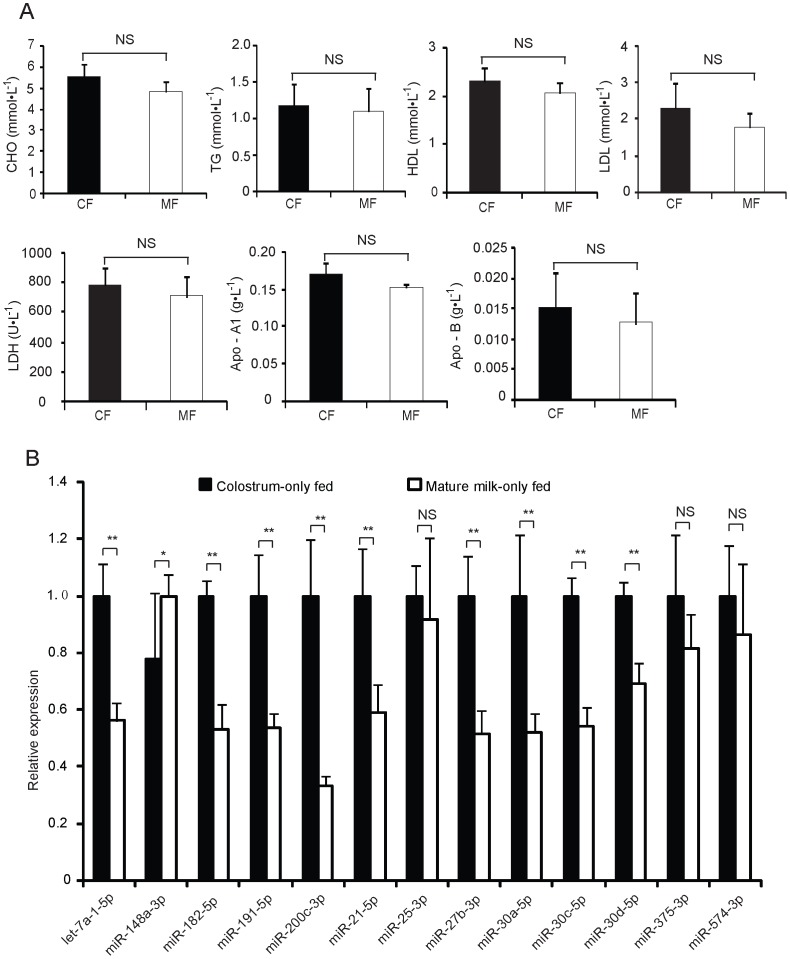
Differences of circulating metabolic indicators and immune-related miRNAs in serum between the colostrum-only and the mature milk-only fed piglets. (A) Seven representative circulating indicators of metabolism. (B) Thirteen well-characterized immune-related miRNAs. The data are normally distributed (Kolmogorov-Smirnov test, *p*>0.05). The statistical significance was calculated by Student’s *t*-test (*n* = 6 per group). Values are means±SD. CF: colostrum-only feeding; MF: mature milk-only feeding.

As shown in Figure 5A, the colostrum-only and mature milk-only fed piglets have similar levels of circulating metabolic indicators in their serum (*p*>0.05). Nonetheless, out of 13 immune-related mRNAs, 12 miRNAs exhibited higher abundance in the colostrum-fed piglets compared with the mature milk-fed piglets (*p*<0.01) (Fig. 5B). The immune-related miRNAs are present in higher concentrations in the colostrum compared with the mature milk (Fig. 4), and the mammalian newborn displays generalized hypofunction of its inflammatory and immune mechanisms. Therefore, it is intriguing to speculate that the these immune-related miRNAs in milk exosomes may be transferred into the infant body via the digestive tract, and may play a critical role in the development of the immune system in infants.

## Discussion

This study reports the comprehensive lactation-related miRNA expression profiles of porcine breast milk exosomes, generated using a deep sequencing approach. We found that immune-related miRNAs are present and enriched in breast milk exosomes and are generally resistant to relatively harsh conditions. These immune-related miRNAs are present in higher numbers in the colostrum compared with the mature milk or the blood of colostrum-only fed piglets compared with the mature milk-only fed piglets.

The presence and enrichment of immune-related miRNAs in breast milk exosomes, especially in the colostrum, was demonstrated. These miRNA-loaded exosomes in breast milk may be transferred into the infant body via the digestive tract. These observations call for further detailed investigations to obtain a thorough understanding of the essential effects of breast milk in the development of the infant’s immune system, especially for the functional roles of miRNA-loaded exosomes. Our findings also highlight a new field of research: the transfer of mammalian genetic material as miRNA from the mother’s milk to the infant via the digestive tract, not through sexual reproduction.

A recent study showed that exogenous plant miRNAs in food are present in the sera and tissues of various animals, and can regulate the expression of target genes in mammals [36]. It will be interesting to distinguish that these functional, specific nucleic acids that are present in the infant’s body (i.e. body fluids and solid tissues) are exogenetic (i.e. mother’s milk-derived, as well as other food-derived) or autologous, which will be beneficial in further deciphering the molecular mechanism underlying the delivery of the food-derived nucleic acids.

This study also highlights that the pig is an ideal model for studying breast-feeding medicine and immune diseases. The pig model offers the advantages of the same general physiology as humans, low genetic variance, and a homogeneous feeding regime. The porcine immune system more closely resembles that of humans for >80% of analyzed parameters, whereas mice were more similar to humans in <10% [37]. For the most part, all the immune cell populations identified in humans and mice are present in pigs [38]. Pig is multiparous (∼10–12 piglets per litter), providing sufficient samples for the present, and other, analysis. Of equal importance, the molecular understanding of pig breast-feeding biology in the development of the piglet’s immune system will improve the economic benefits in the pig industry.

## Materials and Methods

### Ethics Statement

The Yorkshire pigs used in this experiment were obtained from the Sichuan Animal Science Academy, Chengdu, Sichuan, China. Experiments were performed according to the Regulations for the Administration of Affairs Concerning Experimental Animals (Ministry of Science and Technology, China, revised in June 2004) and approved by the Institutional Animal Care and Use Committee in College of Animal Science and Technology, Sichuan Agricultural University, Sichuan, China, under permit No. DKY-B20100805. Animals were allowed access to feed and water *ad libitum* under normal conditions.

### Breast Milk Collection

Three healthy Yorkshire sows (700±5 days old, second birth) were used in this study. Porcine breast milk samples (5–10 ml) were collected from each sow at six lactigenous stages (0, 3, 7, 14, 21 and 28 days after birth). These time points cover the entire porcine lactation period used in the pig industry, from newborn (0 days) up to 28 days, when the piglets are weaned simultaneously. All samples were stored at −80°C until analyzed. All sows had full-term vaginal deliveries of healthy, normal birth-weight piglets.

### AFM

The porcine breast milk exosomes for AFM analysis were prepared as described by Palanisamy et al. [14], with minor modifications. In brief, 5 ml of breast milk was centrifuged at 2500×g for 10 min, twice, to remove cell and fat globules. The supernatant was centrifuged at 12,000×g for 30 min at 4°C to remove any cellular debris. The supernatants were then centrifuged at 120,000×g for 4 hours, and the pellet was resuspended in 10 ml of PBS. The exosome solutions were diluted 1∶500 in deionized water and adsorbed to freshly cleaved mica sheets for 10 min. The redundant solution was removed by careful absorption with a filter paper, and the mica was further dried before detection. Surface morphology was examined under an atomic force microscope (Asylum Research MFP-3D-Bio, Digital Instruments Inc., Santa Barbara, CA) using identical conditions [14]. The size of the exosomes was determined from a line profile of the AFM image.

### Enrichment of Exosomal RNA in Breast Milk

RNA in the breast milk exosomes was isolated as previously described [10]. In brief, raw milk (1.5 ml) was centrifuged at 2,000×g for 10 min to remove fat globules. The supernatant was centrifuged at 12,000×g for 30 min and further filtered through a 0.45 µm PVDF filter to eliminate cells and cellular debris. Approximately 1 ml of the supernatant was mixed with 500 µl of ExoQuick Exosome Precipitation Solution (SBI, CA, USA) and incubated at 4°C for 12 hours. The ExoQuick/supernatant mixture was centrifuged at 1,500×g for 30 min to obtain a beige exosome pellet, which was then re-suspended in 250 µl nuclease-free water. Total RNA from the exosomes was extracted using TRIzol-LS (Invitrogen, CA, USA), following the manufacturer’s instructions. Small RNAs were analyzed with the Agilent Bioanalyzer 2100 and the RNA 6000 Nano LabChip Kit (Agilent, CA, USA).

### Small RNA Sequencing

Total RNA isolated from an individual sow at five stages (3, 7, 14, 21, and 28 days after birth) were pooled in equal quantities for each stage. To evaluate the variations among individuals, the total RNA from three sow’s milk at 0 days were used for library construction, as in our previous descriptions [10]. Eight libraries were subjected to single-end sequencing in 36 nt reads using an Illumina Genome Analyzer II. The bioinformatics pipeline for miRNA discovery and profiling was carried out as previously described, with some improvements [10], [15]. All reads were counted and the identical reads were combined into a single kind. The raw reads were then subjected to a series of additional strict filters (such as the digital filters of base-call quality, reads length, and sequence comparison) with acceptance criteria derived from the statistics of mammalian miRNAs in miRBase 18.0. The reads originated from porcine known classes of RNAs (i.e., mRNA; rRNA, tRNA, snRNA, and snoRNA; and repetitive sequence elements) were also filtered. In addition, to ensure higher reliability of the reported results, only the reads that were observed more than 10 times for each library were used in subsequent analyses. The raw reads that passed through a series of filters were called “mappable reads”, and these were mapped to the 228 pig and 1,527 human pre-miRNAs registered in miRBase 18.0 (November 2011), and the pig genome (∼2.26 Bbp) (Sscrofa9, ftp://ftp.sanger.ac.uk/pub/S_scrofa/assemblies/), using NCBI Local BLAST. The small RNA sequence data discussed in this publication have been deposited in NCBI’s Gene Expression Omnibus and are accessible through GEO Series accession number GSE36590.

### Q-PCR

The changes in abundance of 13 immune-related miRNAs over six lactigenous stages were determined by an EvaGreen–based q-PCR approach using a High-Specificity miRNA qRT-PCR Detection Kit (Stratagene, La Jolla, USA) on the CFX96™ Real-Time PCR Detection System (Bio-Rad, CA, USA). Porcine U6 snRNA, 5S rRNA, and Met-tRNA were simultaneously used as endogenous control genes. The primer sequences are shown in Table S1. All measurements contained a negative control (no cDNA template), and each RNA sample was analyzed in triplicate. Relative expression levels of objective miRNAs were calculated using the ^ΔΔ^Ct method.

### Harsh Treatments of miRNAs and q-PCR

Four exogenous miRNAs (cel-lin-4-5p, cel-miR-2-3p, and cel-miR-39-5p of *C. elegans*, and ath-miR-159a-3p of *A. thaliana*), which have no sequence similarity to pig miRNAs, were chemically synthesized. To determine the resistance and stability of three exogenous miRNAs, cel-lin-4-5p, cel-miR-2-3p and cel-miR-39-5p were added directly to raw milk and subjected to eight types of treatment: incubation at (A) 4°C and (B) 26°C for 0.5, 1, 2, 4, 8, or 24 hours; (C) incubation at 70°C for 10, 60, or 300 seconds; (D) incubation at 100°C for 10, 60, or 300 seconds; (E) treatment in pH 1 solution for 5, 30, 60, or 120 minutes; (F) treatment in pH 5 solution for 5, 30, 60, or 120 minutes; (G) subjected to six freeze-thaw cycles at 4°C; and (H) treatment with RNase A (0.16 µg µL^−1^) and RNase T1 (0.4 U µL^−1^) (Fermentas, Shenzhen, China) for 5, 30, 60, 120, minutes at 37°C. The exogenous control genes are unstable under the above harsh conditions; therefore, to normalize inter-sample variations in the RNA isolation step, ath-miR-159a-3p was added to the raw milk after the addition of TRIzol-LS (Invitrogen, CA, USA) after these treatments, which contains RNase inhibitors. The q-PCR was performed as per the above descriptions. The primer sequences are shown in Table S1.

### Measurements of the Colostrum-only and the Mature Milk-only Fed Piglets

Twelve newborn female piglets of three Yorkshire sows, which were used for breast milk collection (four piglets for each sow), were randomly divided into two groups. For the control group, six piglets (two piglets for each sow) were under colostrum feeding from their birth mothers. For the fosterage group, six piglets (two piglets for each sow) were fostered to another three Yorkshire sows after the first 8 days after birth (i.e. mature milk-only feeding). The venous blood (5 ml) of the 12 female piglets was separately collected at 4 days of age (8∶00 A.M). The total RNA from the blood was extracted using TRIzol-LS (Invitrogen), following the manufacturer’s instructions. The whole blood was immediately centrifuged at 1,800 g for 10 minutes at RT, and the resultant serum was stored at −80°C. Serum concentrations of total cholesterol (CHO), triglycerides (TG), high density lipoprotein (HDL), low density lipoprotein (LDL), lactate dehydrogenase (LDH), apolipoprotein A-1 (Apo-A1), and apolipoprotein B (Apo-B) were determined in triplicate for each pig using a Hitachi 7180 automatic analyzer (Hitachi, Tokyo, Japan) and based on the standard enzymatic procedures. The abundances of 13 immune-related miRNAs were measured using the q-PCR as described above.

## Supporting Information

Figure S1 **Length distribution and frequency (%) of mappable reads.**
(DOC)

Figure S2 **Stability of milk-derived miRNAs under various harsh conditions.**
(DOC)

Table S1 **Primer sequences of the q-PCR experiments.**
(DOC)
