# Supplementary material for: Lactation-Related MicroRNA Expression Profiles of Porcine Breast Milk Exosomes
Source: PLoS One. 2012 Aug 24;7(8):e43691. doi: 10.1371/journal.pone.0043691 (PMC3427246; doi:10.1371/journal.pone.0043691)
Supplement: Figure S1 — Length distribution and frequency (%) of mappable reads. (DOC) [file pone.0043691.s001.doc]

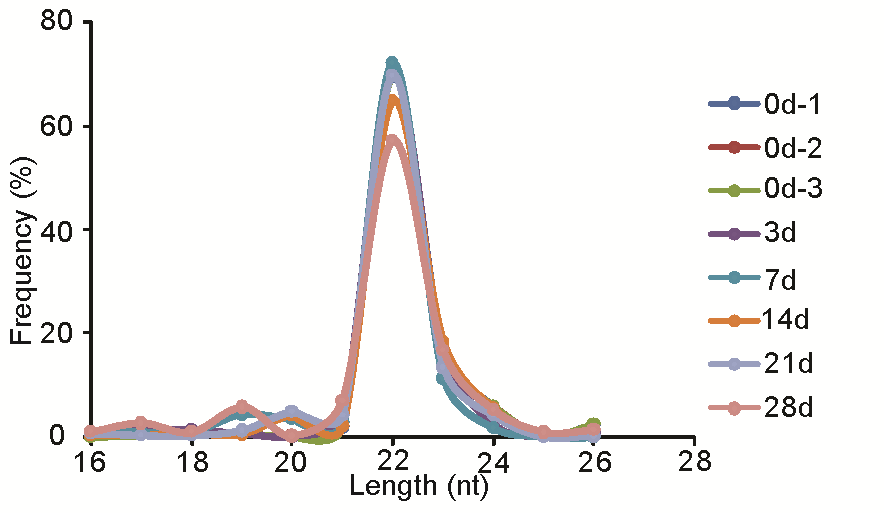


**Figure S1 Length distribution and frequency (%) of mappable reads.** The three biological replicates at 0 days are denoted by 0d-1, -2 and -3, respectively.
