# Supplementary material for: Lactation-Related MicroRNA Expression Profiles of Porcine Breast Milk Exosomes
Source: PLoS One. 2012 Aug 24;7(8):e43691. doi: 10.1371/journal.pone.0043691 (PMC3427246; doi:10.1371/journal.pone.0043691)
Supplement: Figure S2 — Stability of milk-derived miRNAs under various harsh conditions. (DOC) [file pone.0043691.s002.doc]

**
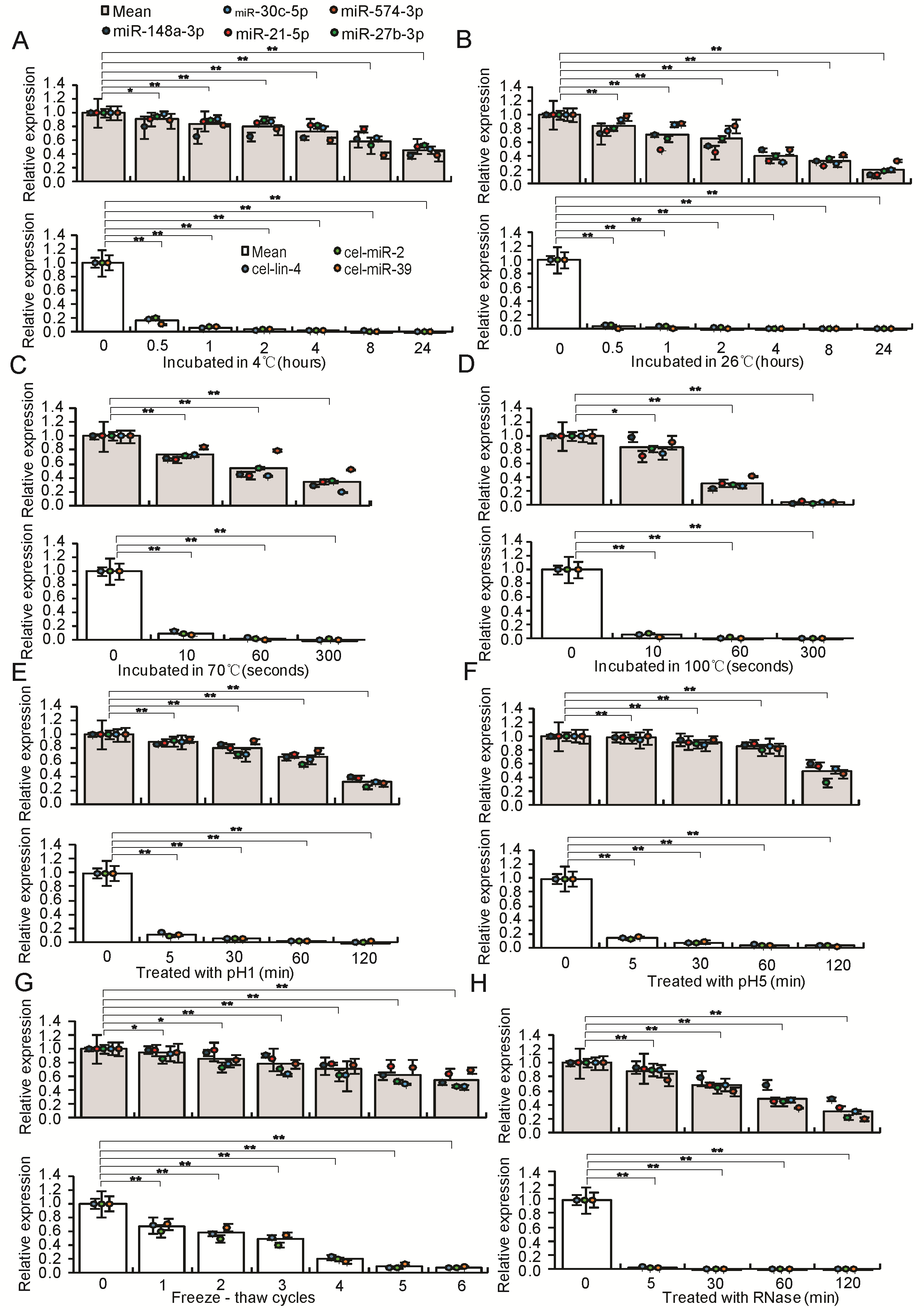
**

**Figure S2 Stability of milk-derived miRNAs under various harsh conditions.** The changes in abundance of four spiked-in *C. elegans* miRNAs and of the five highly expressed immune-related miRNAs under various harsh conditions. Total RNA was extracted and then analyzed by q-PCR. Breast milk was incubated at (A) 4°C and (B) 26°C for 0.5, 1, 2, 4, 8, or 24 hours; (C) incubated at 70°C for 10, 60, or 300 seconds; (D) incubated at 100°C for 10, 60, or 300 seconds; (E) treated in pH 1 solution for 5 , 30 , 60, or 120 minutes; (F) treated in pH 5 solution for 5 , 30 , 60, or 120 minutes; (G) subjected to six freeze-thaw cycles at 4°C; and (H) treated with RNase A and T1 for 5, 30, 60, 120, minutes at 37°C. The data are normally distributed (Kolmogorov-Smirnov test, *p* > 0.05). The statistical significance was calculated by Student’s *t*-test (**p* < 0.05, ***p* < 0.01). Values are means ± SD.
