# Supplementary material for: Lactation-Related MicroRNA Expression Profiles of Porcine Breast Milk Exosomes
Source: PLoS One. 2012 Aug 24;7(8):e43691. doi: 10.1371/journal.pone.0043691 (PMC3427246; doi:10.1371/journal.pone.0043691)
Supplement: Table S1 — Primer sequences of the q-PCR experiments. (DOC) [file pone.0043691.s003.doc]

**Supplemental Table S1** Primer sequences of the q-PCR experiments

| New Name | Primer | Primer sequence (5' - 3') |
| --- | --- | --- |
| cel-miR-39-5p | FW Primer | AGCTGATTTCGTCTTGGTAATA |
| cel-lin-4-5p | FW Primer | TCCCTGAGACCTCAAGTGTGA |
| cel-miR-2-3p | FW Primer | TATCACAGCCAGCTTTGATGTGC |
| ath-miR-159a | FW Primer | TTTGGATTGAAGGGAGCTCTA |
| let-7a-1-5p | FW Primer | TGAGGTAGTAGGTTGTATAGTT |
| miR-148a-3p | FW Primer | TCAGTGCACTACAGAACTTTGT |
| miR-182-5p | FW Primer | TTTGGCAATGGTAGAACTCACACT |
| miR-191-5p | FW Primer | CAACGGAATCCCAAAAGCAGCT |
| miR-200c-3p | FW Primer | TAATACTGCCGGGTAATGATGGA |
| miR-21-5p | FW Primer | TAGCTTATCAGACTGATGTTGAC |
| miR-25-3p | FW Primer | CATTGCACTTGTCTCGGTCTGA |
| miR-27b-3p | FW Primer | TTCACAGTGGCTAAGTTCTGC |
| miR-30a-5p | FW Primer | TGTAAACATCCTCGACTGGAAGC |
| miR-30c-1-5p,miR-30c-2-5p | FW Primer | TGTAAACATCCTACACTCTCAGCT |
| miR-30d-5p | FW Primer | TGTAAACATCCCCGACTGGAAGC |
| miR-375-3p | FW Primer | TTTGTTCGTTCGGCTCGCGTGA |
| miR-574-3p | FW Primer | CACGCTCATGCACACACCCACA |
| U6 snRNA | FW Primer | TTATGGGTCCTAGCCTGAC |
|  | RW Primer | CACTATTGCGGGTCTGC |
| 5S rRNA | FW Primer | GCCCGATCTCGTCTGATCT |
|  | RW Primer | AGCCTACAGCACCCGGTATT |
| Met-tRNA | FW Primer | CAGAGTGGCGCAGCGGAAGC |
|  | RW Primer | CGATCCATCGACCTCTGGGTTATG |
